# Acute Effects of Ischemic Intra-Conditioning on 30 m Sprint Performance

**DOI:** 10.3390/ijerph191912633

**Published:** 2022-10-03

**Authors:** Krzysztof Fostiak, Marta Bichowska, Robert Trybulski, Bartosz Trabka, Michal Krzysztofik, Nicholas Rolnick, Aleksandra Filip-Stachnik, Michal Wilk

**Affiliations:** 1 Faculty of Physical Education, Gdansk University of Physical Education and Sport, 80-336 Gdansk, Poland; 2 Provita Zory Medical Center, 44-240 Zory, Poland; 3 Department of Medical Sciences, The Wojciech Korfanty School of Economics, 40-065 Katowice, Poland; 4 Faculty of Physical Education and Sport, Charles University, 500 05 Prague, Czech Republic; 5 The Human Performance Mechanic, CUNY Lehman College, Bronx, New York, NY 10468, USA; 6 Institute of Sport Sciences, Jerzy Kukuczka Academy of Physical Education in Katowice, 40-065 Katowice, Poland

**Keywords:** training, testing, performance, running

## Abstract

The present study aimed to evaluate the effects of ischemic intra-conditioning applied during rest intervals on 30 m sprint performance. Thirty-four trained male (*n* = 12) and female (*n* = 22) track and field and rugby athletes volunteered to participate in the study (age = 19.6 ± 4 years; training experience = 5.3 ± 1.9 years). In a randomized and counterbalanced order, participants performed six sets of 30 m sprints under three different testing conditions: without ischemic intra-conditioning, and with ischemic intra-conditioning at 60% or 80% arterial occlusion pressure applied bilaterally before the first trial of the sprint and during the rest periods between all sprint trials. During experimental sessions, subjects perform 6 × 30 m sprints with a 7 min rest interval between attempts. The cuffs were applied following a 1 min rest period and lasted for 5 min before being released at the 6th minute to allow for reperfusion (1 min + 5 min ischemic intra-conditioning + 1 min reperfusion). The two-way repeated measures ANOVA did not show statistically significant condition × set interaction for time of the sprint (*p* = 0.06; η^2^ = 0.05). There was also no main effect of ischemic intra-conditioning for any condition (*p* = 0.190; η^2^ = 0.05). This study indicates that ischemic intra-conditioning did not enhance the performance of 30 m sprints performed by athletes. However, ischemic intra-conditioning did not decrease performance either.

## 1. Introduction

Blood flow restriction (BFR) is a method of reducing or eliminating blood flow to a limb for a specified duration leading to localized muscle ischemia [1]. Ischemia is obtained through a pneumatic tourniquet or elastic cuffs placed at the proximal-most region of upper or lower limbs and may be applied unilaterally or bilaterally. BFR during exercise has been shown to improve acute performance (e.g, power and strength endurance) as well as chronic adaptations (e.g., hypertrophy, maximal strength gains) [2,3,4]. The primary mechanisms that promote muscular development following BFR are thought to include increased metabolic stress, muscle fiber recruitment, cellular swelling, enhanced intramuscular signaling for protein synthesis, and proliferation of myogenic stem cells [5]. It has also been suggested that BFR-induced muscle hypertrophy comprises not only the proliferation of contractile proteins but also enlargement of muscle glycogen stores and increased capillary density [4,6,7,8]. Therefore, the use of BFR as part of physical performance training can be useful in different aspects of conditioning for various sports. BFR can be combined with various types of exercise—such as walking, cycling, running and sprinting. Sprint training combined with BFR is a novel method which can not only increase the strength and hypertrophy response, but also increase maximal oxygen uptake in trained individuals [9]. Abe et al. suggested that increased leg muscle strength following BFR intervention may improve the initial acceleration phase (0–10 m) of sprinting in track and field athletes [2]. Further, Chen et al. showed that that the isokinetic knee flexor strength and concentric hamstring-quadriceps ratio as well as heart rate and muscle activation observed in BFR running warm-up were higher than those observed after the traditional warm-up. Thus, these results justify the possible benefits of using BFR for sprint performance [10].

There are four different methods of applying BFR in training as part of physical exercise: pre-conditioning (BFR applied only before a training session), continuous (BFR applied over the exercise as well as over the rest periods), intermittent (applied over exercise, and not applied over the rest periods), and ischemic intra-conditioning (applied during rest periods between exercise) [11]. The most common and most frequently used method is continuous BFR. Previous studies indicate that BFR used only during exercise increases acute as well as chronic muscle adaptations. Gepfert et al. [12] showed an immediate increase in power and strength-endurance capacity, as well as improvements in 1RM testing during the bench press exercise following BFR [12]. Behringer et al. [13] noted the benefits of training with BFR during sprint training, producing decreased levels of muscle damage, greater increases in rectus femoris muscle thickness, and a higher rate of force development in the BFR compared to the control condition [13]. However, frequent BFR exercise applied in the same muscle area can impair muscle structure in the region under the cuff, increasing risk not only for sports performance but for the health of the athlete [14,15]. Furthermore, with high pressures, BFR exercise frequently produces greater perceptual demands than free-flow exercise performed at the same intensity, making implementation of training tasks difficult [16]. However, out of the four training methods, ischemic intra-conditioning is the least researched while still showing beneficial effects without interfering with the main movement during exercise. In a prior study, ischemic intra-conditioning (BFR applied during rest periods only) caused a significant increase in power output and bar velocity during the bench press exercise at 60% 1RM (5 sets of 3 repetitions) [1]. Similar ischemic intra-conditioning protocols used on lower limbs caused a smaller decline in power output during sets 3 to 5 compared to the control condition [11]. Thus, ischemic intra-conditioning applied during rest periods can be an effective tool to induce or maintain a high level of power capacity by preventing or reducing increasing exercise fatigue. However, all studies on the effect of ischemic intra-conditioning have been carried out during resistance training, leaving no data on the effect of this BFR application in other types of exercise—such as walking, running, sprinting and cycling.

It is without question that running speed is an essential component of most sports. Although numerous aspects of sprint training have already been investigated, there is little knowledge about the impact of BFR on sprint performance. Cook et al. reported that the 40 m sprint time in elite rugby players improved to a larger extent after BFR resistance training applied only during the exercise (e.g., intermittent BFR) when compared to the control conditions [3]. Behringer et al., showed the benefits of sprint training with BFR (6 × 100 m sprints at 60–70% of their maximal 100 m sprint) on sprint performance [13]. However, it is important to consider that BFR may impair sports activity techniques, and it is unknown whether submaximal or maximal sprinting with BFR on the lower limbs affects their motion (movement technique, rhythm and length of running steps). Therefore, the use of BFR during the rest periods seems to be an optimal solution that copes with the potential risks and negative consequences such as pain and discomfort that occur when using BFR during exercise. However, to the best of the authors’ knowledge, there has been no research to-date that has examined the effects on physical performance of BFR applied only during rest intervals between sprints.

Given the use of BFR as a means of developing strength and power of the lower limbs, it would be interesting to investigate whether sprint training with ischemic intra-conditioning affects sprint time performance. Because previous studies have shown that ischemic intra-conditioning increases power performance or prevents fatigue during later sets of resistance exercise, it can be assumed that a similar effect may be obtained during sprint training. To the best of our knowledge, there is no available data regarding the acute effects of ischemic intra-conditioning on sprint performance. Since the 30 m sprint is a basic exercise performed in many sports, the present study aimed to evaluate the effects of ischemic intra-conditioning applied during rest intervals on 30 m sprint performance. We hypothesized that the application of ischemic intra-conditioning improves sprint performance during successive 30 m trials.

## 2. Materials and Methods

### 2.1. Experimental Approach to the Problem

**Figure 1:**
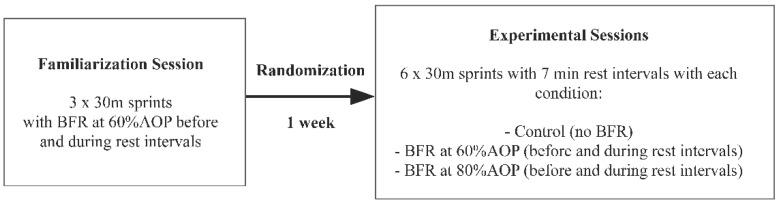
CONSORT flow diagram.

The study was performed with a crossover, randomized design. Each subject participated in three experimental sessions, and each experimental session was performed one week apart: (a) ischemia with a 60% AOP (total arterial occlusion pressure) applied before each trial of sprints; (b) with BFR applied at 80% AOP before each trial of sprints; (c) control condition, when the ischemia was not applied. During each session, participants performed sprints 6 × 30 m with a 7 min rest period between each sprint (sprint from a standing starting position). In each of the ischemic conditions, two 10 cm-wide cuffs were applied bilaterally to the most proximal region of the thighs of each participant before the first set of sprints and during all rest intervals between the sprints. The cuffs were inflated to either 60% AOP or 80% AOP and applied for 5 min beginning one minute after the sprint trial and removed one minute before the subsequent trial to allow for reperfusion (1 min passive rest + 5 min BFR + 1 min reperfusion/passive rest). The control session did not use ischemia (Figure 1). All stages of the study were performed at the University of Physical Education and Sport in Gdansk, Poland.

### 2.2. Subjects

Thirty-four trained male (*n* =12) and female (*n* =22) track and field sprinters and jumpers as well as rugby players volunteered for the study (age = 19.6 ± 4 years; body mass = 66.0 ± 9.4 kg; height = 173.5 ± 9.7 cm; training experience = 5.3 ± 1.9 years). The inclusion criterion were: (a) minimum 3 years of experience in professional sport training, with minimum 3 training sessions per week; (b) free from musculoskeletal injuries for at least 6 months before the study; (c) age under 18 years old. The subject followed their standard diet throughout the entire duration of the experiment. Further, the subjects did not use additional supplements or drugs. Before the beginning of the experimental sessions, the subjects were informed and were aware of the potential risks of participating in the research and they signed written informed consent. The experimental project was approved by the Academy of Physical Education Bioethics Committee for Scientific Research (02/2019), in accordance with the ethical standards of the Declaration of Helsinki, 1983. No participants withdrew from the study.

### 2.3. Procedures

#### 2.3.1. Familiarization Session

One week before the main experiment, the participants performed a familiarization session. During the familiarization session, the subjects performed a general warm-up followed by 3 trials of 30 m sprints with BFR applied at a pressure of 60% AOP. The BFR was applied before the first trial and during the two rest intervals in a similar manner to that used in the experimental sessions below.

#### 2.3.2. Experimental Sessions

In a randomized and counterbalanced order, participants performed six sets of 30 m sprints under 3 different testing conditions: (a) ischemia with a 60% AOP (total arterial occlusion pressure) applied before each trial of sprints; (b) with BFR applied at 80% AOP before each trial of sprints; (c) control condition, when the ischemia was not applied. During each experimental protocol, each participant performed 6 sets of 30 m sprints with 7 min rest intervals between set. Each experimental session was performed with seven days’ rest periods. The warm-up performed before the main experimental part was similar to that performed in the familiarization session. A Sectro Timing Systems TS-L2 chronometer was used for the evaluation of sprint time, where data received from photocells allow precise control of the training intervention. Measurements were made independently for each sprint trial. The evaluations were carried out on an indoor synthetic six-lane track. The research team was blinded to the group allocation of each participant. The order of conditions was chosen randomly and in a counterbalanced order using a free online randomization program (randomization.com). All participants completed the described testing protocol that was carefully replicated in subsequent experimental sessions.

#### 2.3.3. Ischemic Intra-Conditioning Procedure

During the BFR conditions, the cuffs were applied bilaterally as high as possible on the proximal portion of each thigh. For this experiment, 10 cm-wide FitCuffs were used (FitCuffs, Denmark). To determine each participant’s arterial occlusion pressure (AOP), a handheld Doppler was used (Edan SD3, Sonoline C doppler with 8 MHz probe, Contec, China). The researcher gradually inflated the cuffs by 50–70 mm Hg every 10 s until full occlusion, and then the pressure where the Doppler ultrasound was no longer being auscultated was determined as the participant’s AOP. The cuffs pressure for ischemia was set to ~60% or ~80% AOP (110 ± 13 mm Hg; 147 ± 18 mm Hg, respectively). For the ischemic intra-conditioning groups, 5 min of BFR was applied 1 min after the sprint trial, and released 1 min prior to the start of the subsequent sprint trial to allow for limb reperfusion (1 min after sprint + 5 min of ischemia + 1 min reperfusion).

#### 2.3.4. Statistical Analysis

All statistical analyzes were performed using Statistica 9.1. Results are presented as means with standard deviations. The Shapiro–Wilk test was used to verify normality, homogeneity, and sphericity of the sample data variances, respectively. Differences between the BFR and control conditions were examined using two-way repeated measures ANOVA [3 conditions (BFR at 60% AOP vs. BFR at 80% AOP vs. control) × 6 sprint trials]. Partial eta squared were determined for main effects and interactions (small = 0.01–0.059; moderate = 0.06–0.137; large > 0.137). Tukey’s post hoc test was used to locate the differences. The ES Cohen’s d was used for pairwise comparisons (large d > 0.8; moderate d 0.8–0.5; small d 0.49–0.20; trivial d < 0.2). Furthermore, two-way repeated measures ANOVA was performed for the delta values between sprint trials (Sprint no. 2—Sprint no. 1; Sprint no. 3—Sprint no. 1; Sprint no. 4—Sprint no. 1; Sprint no. 5—Sprint no. 1; Sprint no. 6—Sprint no. 1) for all conditions.

## 3. Results

The ANOVA (2-way repeated measures) did not show statistically significant condition × set interaction for time of sprint (conditions × sets; *p* = 0.06; η^2^ = 0.05; Table 1). There was also no main effect of BFR for any condition (*p* = 0.190; η^2^ = 0.05). The ES for the two experimental conditions for all measured variables are presented in Table 2. The two-way ANOVA for delta values between sprint trials showed statistically significant condition × set interaction for time of sprint (conditions × sets; *p* = 0.016; Table 3); however, the post hoc did not show statistically significant differences between conditions. There was also no main effect in delta values of BFR for any condition.

## 4. Discussion

The main finding of the study was that ischemic intra-conditioning did not alter the time of 30 m sprints in competitive athletes. The lack of significant differences compared to the control condition was observed when we applied BFR at a pressure of both 60% AOP and 80% AOP. However, what is particularly significant is that despite no differences in sprint time during all 6 trials, ischemic intra-conditioning did not decrease performance in successive sprints either. Lack of increased speed performance during BFR conditions compared to the increase power performance during BFR bench press performance [1] can be related with the muscle area where BFR was used, as well as the type of movement. Gepfert et al. showed that to induce positive changes following BFR, the lower limbs need much higher cuffs pressure compared to the lower limbs [17]. Therefore, the acute effect of ischemic pressure on physical performance can be related to the area of muscle where ischemia is applied, and its effect may be different in the upper and lower limbs [11]. It is also speculated that thigh circumference or composition of the limbs may directly impact on the restriction of flow within individuals, which may account for some of the variability in the response to BFR [18,19,20]. BFR potentially increases metabolic stress and muscle fiber recruitment and enhances intramuscular signaling for protein synthesis, all of which are important factors thought to positively influence muscle adaptation [5]. This study suggests that ischemic intra-conditioning between sprints, although not increasing efficiency, could be an additional tool to increase acute physiological responses by the metabolic stress increases, muscle fiber recruitment, and enhanced intramuscular signaling for protein synthesis—without losing sprint training efficiency. However, the conducted research did not assess the acute metabolic and hormonal responses that would support this assumption.

To the best of the authors’ knowledge, the present study is the first to evaluate the effects of ischemic intra-conditioning used between 30 m sprints. So far, this method of applying BFR has been used during resistance exercises. It has been shown that ischemic intra-conditioning between sets of resistance exercises may be a significant factor in increasing the acute level of power performance. Wilk et al. showed that ischemia applied only during the rest intervals increased power capacity during the resistance exercise compared to the control one (bench press; 5 sets; load of 60% 1RM) [1]. Interestingly, the increase in performance for athletes who used ischemic intra-exercise was observed mainly in sets 3 to 5 which suggests that this model of applied BFR (only during the rest periods) allows athletes to maintain a certain exercise capacity despite increased fatigue in working muscles [1]. The effect of maintaining explosive performance during progressive fatigue following ischemic intra-conditioning was also observed in a study by Trybulski et al. who showed that ischemic intra-conditioning applied to the lower limbs does not increase explosive performance during a squat exercise at 60% 1RM [11]. However, despite no enhancement in power performance, the ischemia applied during the rest period between sets prevented progressive fatigue [11]. Therefore the ischemia applied only in rest intervals between successive efforts could potentially be an additional tool to increase or maintain performance during multi-set resistance training sessions.

Maintenance of sprint efficiency due to ischemia may be associated with physiological factors similar to those observed with pre-conditioning ischemia. It has been hypothesized that tissues previously exposed to BFR become more resistant to the effects of ischemia during exercise and exercise-induced fatigue [21]. There is significant evidence that acute application of BFR that induces localized tissue ischemia may exert a positive effect on skeletal-muscle function, increasing exercise capacity. Ischemia used as pre-conditioning influences changes in systemic VO2, and in deoxygenation of muscle which increases the energy stocks [22,23,24,25,26,27,28,29]. Furthermore, considering the process of chronic muscle adaptation, the results of Torma et al. may also indicate significant benefits of the ischemic intra-conditioning application even if such an intervention does not increase physical performance [30]. Torma et al. showed that ischemic intra-conditioning may impact gene expression of angiogenesis, thereby affecting the timing of muscle recovery as well as muscle hypertrophic responses [30,31]. In this connection, the ischemic intra-conditioning can impact acute physical changes as observed by Wilk et al. and Trybulski et al. but can also stimulate chronic muscle changes [1,11]. Further, Taylor et al. showed that post-exercise application of BFR could enhance physiological responses such as maximal oxygen uptake (e.g., VO2 max) compared to control conditions in as little as 8 training sessions over 4 weeks [32].

In addition to possible physiological responses following ischemic intra-conditioning, it should be noted that this method of BFR has potentially important practical implications and benefits. The application of BFR during exercise subjectively causes elevated muscle pain and exertion [33], potentially reducing its use in elite athletes’ secondary to heightened perceptual demands [34]. Further disadvantages mainly concern the potential attenuation of muscle growth in the area underneath the occlusive cuff, especially when the cuff is narrow [14,15]. Kacin and Strazar [15] showed reduced cross-sectional area and a tendency for reduced hypertrophy at the level of cuff application, suggesting that high compression and shear stress under the cuff may have a deleterious impact on muscle tissue—although their studies used arbitrary (e.g., 230 mm Hg) and not personalized pressures (e.g., a percentage of AOP). Given that such a negative effect was not observed after exercise with lower inflation pressures [35], it appears that high cuff pressures (>230 mm Hg) was the key negative factor. However, the application of BFR only during the rest intervals eliminates both the negative effects described above and the risk associated with frequent use of BFR during exercise (e.g., higher perceptual demands and/or attenuated muscle growth directly under the cuff). Therefore, coaches and athletes may prudently use ischemic intra-conditioning instead of continuous BFR applications to increase acute and chronic adaptations thought to enhance performance in athletes while minimizing the risk of excessive muscle damage under compression [36,37]. Although tissue hypoxia is likely to be the key stressor of cellular metabolism during BFR exercise, the influence of long-term ischemic intra-conditioning on muscle oxygen kinetics remains unknown.

It should be assumed that despite no differences in kinematic variables between conditions in this study, ischemic intra-conditioning potentially caused a significant increase in physiological, hormonal, and metabolic post-exercise responses. The increase in physiological and metabolic stress following ischemic intra-conditioning may cause increased post-exercise fatigue that may affect the duration of post-workout recovery [18,38,39]. Therefore, despite no improvement in sprint performance in our study following the BFR intervention, we may assume that such a training intervention may have a beneficial effect on acute metabolic and hormonal responses. However, in the present study, the physiological and metabolic responses were not determined on an acute or chronic basis and are the main limitations that should be addressed in future studies. A further limitation of this study was the lack of measurement of the REP scale and pain, especially since the subjects reported symptoms of discomfort while resting under BFR. In addition, further research should compare the effects on performance changes of BFR applied only during the rest periods between different training protocols and types of exercise as well as BFR duration.

## 5. Conclusions

This study did not show an improvement in 30 m sprint when ischemic intra-conditioning was used. However, despite no enhancement in sprint performance, ischemic intra-conditioning prevented progressive declines similar to the control condition. Therefore, maintaining sprint performance following ischemic intra-conditioning may influence chronic muscle adaptations without sacrificing acute sports performance. The ischemic intra-conditioning intervention potentially increased metabolic stress, muscle fiber recruitment, and enhanced intramuscular signaling for protein synthesis—all significant factors thought to mediate positive muscle adaptation.

## Figures and Tables

**Table 1:** Difference in performance variables during control and BFR conditions.

	CONTROL [s] (95% CI)	BFR 60% AOP [s] (95% CI)	BFR 80% AOP [s] (95% CI)	Interaction Condition × Sprint Trial	Main effect of BFR Condition
Sprint no. 1	4.46 ± 0.33 (4.34 to 4.57)	4.48 ± 0.30 (4.37 to 4.58)	4.45 ± 0.30 (4.34 to 4.56)	0.052	0.190
Sprint no. 2	4.44 ± 0.35 (4.32 to 4.56)	4.47 ± 0.32 (4.36 to 4.58)	4.44 ± 0.32 (4.33 to 4.55)		
Sprint no. 3	4.48 ± 0.34 (4.36 to 4.59)	4.46 ± 0.35 (4.34 to 4.58)	4.44 ± 0.33 (4.33 to 4.56)		
Sprint no. 4	4.44 ± 0.32 (4.33 to 4.55)	4.46 ± 0.34 (4.34 to 4.58)	4.47 ± 0.32 (4.35 to 4.58)		
Sprint no. 5	4.47 ± 0.32 (4.36 to 4.58)	4.51 ± 0.33 (4.39 to 4.62)	4.48 ± 0.32 (4.37 to 4.59)		
Sprint no. 6	4.44 ± 0.34 (4.32 to 4.56)	4.46 ± 0.36 (4.33 to 4.58)	4.48 ± 0.30 (4.37 to 4.58)		

All data are presented as mean with standard deviation [SD]; CI = confidence interval; no. = number; BFR = blood flow restriction; AOP = arterial occlusion pressure.

**Table 2:** Differences in effect size between control and BFR conditions.

	CONTROL vs. BFR 60% AOP	CONTROL vs. BFR 80% AOP	BFR 60% AOP vs. BFR 80% AOP
Sprint no. 1	0.06	0.03	0.10
Sprint no. 2	0.09	0.00	0.09
Sprint no. 3	0.06	0.12	0.06
Sprint no. 4	0.06	0.09	0.03
Sprint no. 5	0.12	0.03	0.09
Sprint no. 6	0.06	0.13	0.06

BFR = blood flow restriction; AOP = arterial occlusion pressure; no. = number.

**Table 3:** A comparison between particular sets of sprint under control and BFR conditions.

	CONTROL [s] (95% CI)	BFR 60% AOP [s] (95% CI)	BFR 80% AOP [s] (95% CI)	Interaction Condition × Delta Sprint	Main Effect of Delta BFR Condition
Sprint no. 2—Sprint no. 1	−0.020± 0.097 (−0.054 to 0.014)	0.016 ± 0.111 (−0.022 to 0.055)	−0.019 ± 0.116 (−0.060 to 0.021)	0.016 *	0.59
Sprint no. 3—Sprint no. 1	0.010 ± 0.136 (−0.037 to 0.057)	−0.016 ± 0.122 (−0.058 to 0.027)	−0.009 ± 0.093 (−0.042to 0.023)		
Sprint no. 4—Sprint no. 1	−0.019 ± 0.133 (−0.065 to 0.028)	−0.017 ± 0.110 (−0.056 to 0.021)	0.026 ± 0.153 (−0.027 to 0.079)		
Sprint no. 5—Sprint no. 1	−0.022 ± 0.116 (−0.062 to 0.019)	−0.008 ± 0.096 (−0.041to 0.026)	−0.008 ± 0.101 (−0.043to 0.027)		
Sprint no. 6—Sprint no. 1	0.015 ± 0.088 (−0.016 to 0.046)	0.030 ± 0.119 (−0.012 to 0.071)	0.026 ±0.080 (−0.002 to 0.054)		

All data are presented as mean with standard deviation [SD]; CI = confidence interval; no. = number; BFR = blood flow restriction; AOP = arterial occlusion pressure; * = Statistically significant differences *p* < 0.05.

## Data Availability

The data presented in this study are available on request from the corresponding author.
